# A rare clinical presentation of acute gouty arthritis

**DOI:** 10.1093/rap/rkaa022

**Published:** 2020-06-29

**Authors:** Svitlana Smiyan, Andriy Lepyavko, Ulyana Slaba, Oleg Slabyy, Roman Komorovsky

**Affiliations:** r1 2nd Department of Internal Medicine, I. Horbachevsky Ternopil National Medical University; r2 ‘Multysono’ Medical Center, Ternopil, Ukraine

**Fig. 1:**
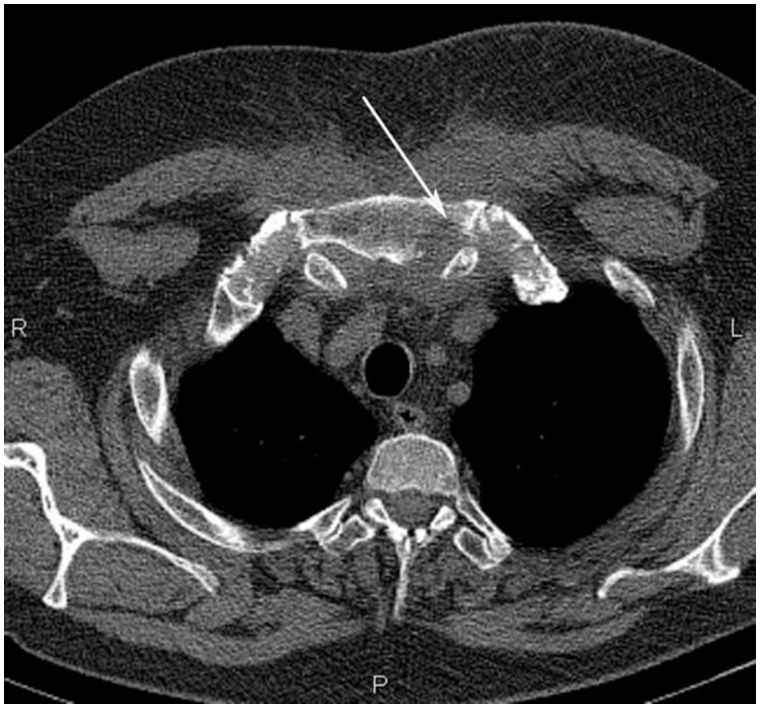
Transverse plane computed tomography image showing signs of destruction of the left sternoclavicular joint (arrow)

A 45-year-old obese male (BMI 40.8 kg/m^2^) presented with acute-onset swelling and severe pain in the left sternoclavicular joint associated with fever (≤38.2°C). His past medical history was notable for controlled hypertension and hypercholesterolaemia. He did not follow any particular diet and admitted occasional alcohol use (less than once a week). Laboratory evaluation showed leucocytosis of 14 200/μl, elevated erythrocyte sedimentation rate of 70 mm/h, hypoalbuminaemia, elevated levels of α_2_- and β-globulins and a decreased albumin-to-globulin ratio. His serum uric acid concentration was 392 μmol/l [6.6 mg/dl; normal range: 202–417 μmol/l (3.4–7.0 mg/dl)]. Computed tomography revealed destructive changes in the left sternoclavicular joint (Fig. 1).

With suspicion for malignancy, the patient was referred to the oncological centre, where resection of the left sternoclavicular joint was performed. Histological analysis, including polarized light microscopy, revealed uric acid crystals in the joint and periarticular tissues consistent with gout.

Acute arthritis of the left sternoclavicular joint is a very rare manifestation of gout, with only a few reports available in literature [1, 2]. Nevertheless, gout should be considered in the differential diagnosis of left sternoclavicular joint swelling, especially in patients with multiple metabolic risk factors. The differential diagnosis may be challenging if serum uric acid concentrations are not elevated. Nonetheless, a normal serum uric acid concentration does not exclude a diagnosis of gout, and gouty attacks are not always related to uric acid concentrations.

*Funding*: No specific funding was received from any funding bodies in the public, commercial or not-for-profit sectors to carry out the work described in this manuscript.

*Disclosure statement*: the authors have declared no conflicts of interest.
